# A Point-of-Care Diagnostic Platform for Detecting Host Immune Activation due to Infections: Cytocapture of Biomarkers *In Situ* (CyBIS)

**Published:** 2025-01-16

**Authors:** Timothy A. McCaffrey, Richard Wargowsky, Kevin Jaatinen, Faisal Al Munajjed, Farris Alqalam, John Perkins, Zane Hayden, Mary Pasquale, Grace Holloway, Jennifer Goldman, Zachary Falk, Tisha Jepson, David Yamane, John LaFleur, Andrew Meltzer, Soroush Shahamatdar, Ryan Heidish, Eduard Shaykhinurov, Aditya Loganathan, Tarun Loganathan, Taylor Bolden

**Affiliations:** 1 Department of Medicine, The George Washington University Medical Center, Washington, DC, United States of America; 2 Department Anesthesiology and Critical Care Medicine, The George Washington University Medical Center, Washington, DC, United States of America; 3 Department of Emergency Medicine, The George Washington University Medical Center, Washington, DC, United States of America; 4 Department of Microbiology, Immunology, and Tropical Medicine, The George Washington University Medical Center, Washington, DC, United States of America

**Keywords:** Infection, Neutrophil, Elastase, CD66b, CD15, Point of care, Sepsis, COVID-19

## Abstract

The prompt diagnosis of internal infections is an important, but surprisingly difficult, component of healthcare. Existing clinical and laboratory tests, such as complete blood counts, are low accuracy (~70%), time consuming (>90 min), and require expensive blood analyzers. More sensitive and specific tests, such as PCR or sequencing, require access to a sample of the infected tissue, genomic data for all potential pathogens and expensive equipment. Culturing organisms introduces its own biases and is prone to false positives due to contamination. We describe a device for the rapid isolation of neutrophils and measurement of neutrophil elastase activity to provide a measure of host immune activity toward a broad range of infectious agents. CyBIS (Cytocapture of Biomarkers *In Situ*) is a Point-of-Care (POC) device that isolates neutrophils from a small volume of blood (75 ul) using anti-CD66b antibody-coated paramagnetic beads and analyzes elastase activity *via* a kinetic assay. This inexpensive and rapid (35 min) testing system provides a quantitative measure that combines neutrophil count with neutrophil elastase activity per cell to diagnose elevated host immune activity and possible infection. Neutrophil-associated elastase is elevated in emergency department patients with clinically relevant infections, including sepsis, compared to healthy control patients.

## INTRODUCTION

Quickly and accurately diagnosing infection in a clinical setting is an important medical need. It is vital to prevent the potential spread of the infection into the bloodstream, or conversely, prevent the unnecessary use of antibiotics when an infection is absent [1]. Current methods however, leave much to be desired. Traditionally, physicians have relied on monitoring host immune activation by measuring overt biosigns, such as fever or an elevated white cell count, but the sensitivity and specificity is quite limited. Newly available point-of-care systems based on PCR can detect pathogen Deoxyribonucleic Acid (DNA) or Ribonucleic acid (RNA), but they are limited to detecting specific pathogens in the panel. There is little doubt that the plummeting cost of Next Generation Sequencing (NGS) could significantly improve the diagnosis of suspected infections, but the intensive laboratory preparations and the need to sample directly from infected tissue or fluid have hindered clinical utility [2]. Currently, NGS is a still too cost and time intensive to be considered an effective first-line diagnostic tool. The purpose of our current work was to create a rapid, inexpensive, and portable system to diagnose medically relevant infections before a patient left their appointment (<1 h). Similar to fever or elevated white count, we planned to utilize the host’s immune system as a biosensor, but we sought to measure objective and quantitative changes at the source, the host’s immune cells. A key advantage of this approach is that we can diagnose indirectly from a small blood sample without the attendant complications of a biopsy of infected tissue. Even for difficult-to-diagnose infections, such as biofilm-forming microbes on implants which may not shed measurable levels of bacteria or bacterial DNA into the bloodstream, the host’s systemic immune response can react in a very sensitive manner. Properly configured, such a system could be used in physician’s offices, urgent care, emergency rooms, and especially in low resource environments such as rural or developing areas. Previously, we have used whole blood RNA to evaluate the expression level of specific mRNAs measuring host neutrophil activation to bacterial, biofilm, and viral infections [3–6]. These markers report both the level of activation and the type of reaction reported by host neutrophils. It was observed that in parallel to the elevated levels of neutrophil activation markers in whole blood RNA, there were increases in the levels of neutrophil elastase in purified neutrophils from patients with infections. Neutrophil elastase, an enzymatic protein produced and released by neutrophils to cleave histones into antimicrobial peptides and Neutrophil Extracellular Traps (NETs), should make an ideal biomarker of neutrophil activation, except it is rapidly bound and neutralized by several protease inhibitors present in plasma [7]. While there are many potential protein or small molecules that are secreted during infections, the host’s essential need to limit signal propagation by binding and neutralizing these factors is a major complication to their accurate measurement in blood. Consequently, our prior studies have shown that even when striking increases in neutrophil elastase are observed in purified neutrophils, there is no significant increase in elastase in plasma of the same patient.

Thus, the current studies describe a device that automates the isolation, preparation, and analysis of live neutrophils from whole blood, thereby minimizing the effects of circulating elastase inhibitors. The design requirements were to quantify neutrophil elastase activity rapidly at the Point of Care (POC), in a ‘load and leave’ format that was inexpensive and used disposable consumables to prevent contamination. The initial results suggest that the immunocapture of neutrophils coupled to elastase activity assay is a viable strategy for detecting the host response to infection. This strategy has broader implications for assaying almost any relevant biomarker in a broad range of cell types for which there are known surface markers and an enzymatic reporter.

In short, Cytocapture of Biomarkers *In Situ* (CyBIS) works by mixing a small volume (75 ul) of blood with antibody-coated paramagnetic beads. Using a magnet and a fluidic system, the unbound extraneous blood components are separated from the bead-bound neutrophils, and are flushed away. A solution that lyses the remaining neutrophils and contains a colorimetric substrate for elastase is then introduced and measured at 1 min intervals by a built-in Light-Emitting Diode (LED) and optical transmittance sensor. From the decreasing optical transmittance, a rate constant is calculated. The machine assay is entirely automated, taking approximately 35 min. to complete. It has been tested with blood from both a standard venipuncture into an Ethylene Diamine Tetraacetic Acid (EDTA) coated blood collection tube as well as a fingerstick.

## MATERIALS AND METHODS

### CyBIS design and build

The CyBIS enclosure, as well as various internal parts and fittings, was custom-designed in ThinkerCAD and Fusion 360, and printed using commercial-grade resin on a FormLabs 3B 3D printer.

#### Enclosure design:

A variety of commercially-sourced power supplies, displays, pumps, valves, sensors, LEDs, filters, tubing and magnets were integrated into this custom housing.

#### Electronic:

Operations were controlled by an Arduino Mega 2450 Micro Controller (AMc) running custom software coded in a variant of C++ (Github). Most of the electrical connections to and from the Arduino AMc to pumps, valves, and sensors were facilitated *via* a custom Printed Circuit Board (PCB). Power was supplied by 5V DC transformers, and regulated on the AMc board.

#### Fluidics:

**Figure 1:**
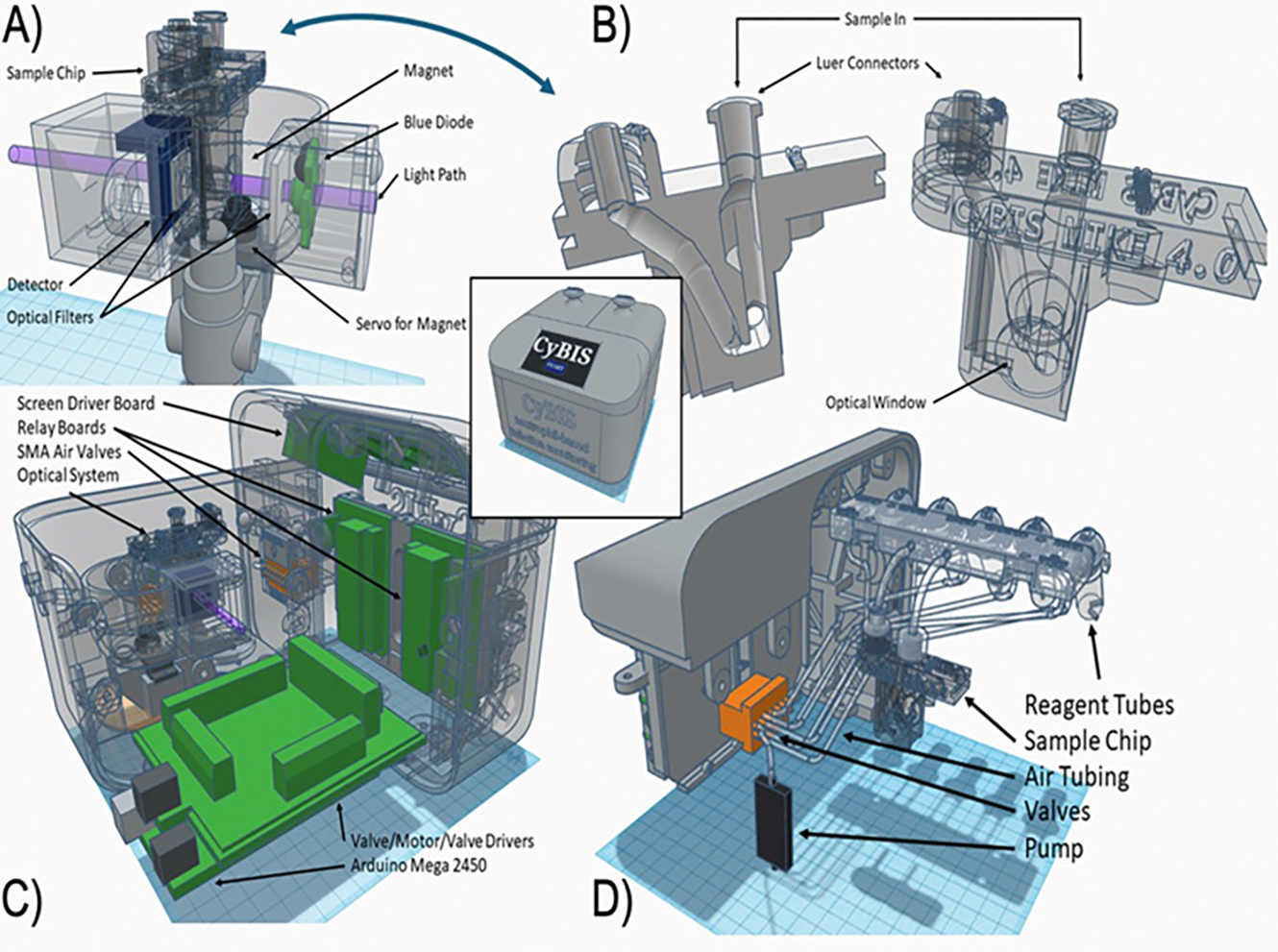
CyBIS overall design: The assembled device is shown in the upper left, (20 cm W × 20 cm D × 15 cm H), with a front panel touch display to control operations and report progress and results.

Fluid handling was powered by a single Bartels MP6 micropump controlled by the AMc. The pump provides positive air pressure to the fluid reservoirs containing the reagents. Air pressure is directed by electrically actuated air valves (Takasogo). For assay steps that requiring gentle mixing, for example the initial incubation of beads and blood, air was pumped directly into the chip. The air intake and vent were fitted with 0.2 micron filters to contain aerosols. The fluid reservoirs are adapted from disposable 2 and 4 ml polypropylene tubes. They can be prefilled and stored separately for ready access. All rinsed blood components and waste reagents are flushed through a single line into a disposable tube, reducing disinfection and cleaning time (Figure 1).

#### CyBIS chip:

The CyBIS chip was 3D printed and serves as a disposable reaction chamber for the blood to interact with paramagnetic beads, undergo a washing procedure and be assayed by absorption of 405 nm light. It is printed in a single piece with Luer connectors for inlet and outlet. A key feature of the chip is the presence of a light path through the collection/reaction chamber. The chamber is closed at each end with a borosilicate glass cover slip sealed to the 3D printed chamber with Ultraviolet (UV) activated resin to create the light path through the fluid and ensure maximum optical transmittance.

#### Optical system:

A sensitive and stable optical absorbance light path was assembled from commercial and custom parts. The LED light source emits in the 405–410 nm range (3 W, 3.5 V, CMPN Inc.) and is powered by an attenuated 5V power line from the AMc. Light intensity is reduced with an ND2 filter prior to the CyBIS chip chamber to prevent excess heat and saturation of the photometer. After passing through the chamber in the CyBIS chip (1 cm light path), the light was filtered with a 410 nm bandpass filter to remove autofluorescent noise before being measured by the Red/Green/Blue (RGB) color sensor (Taos TCS34725) which included an infrared filter. The transmitted light was quantified in the blue (B) channel with 16-bit 0–65000 precision.

#### Autofluorescence:

A major complication in the optical system for detecting substrate cleavage was that 410 nm excitation caused autofluorescence of the neutrophils that increased in intensity over time and could overwhelm the absorbance created by substrate cleavage. This is likely due to the progressive generation of free radicals creating modified proteins and lipids with autofluorescence. Neutrophils are rich in myeloperoxidase which could be a source of this signal [8]. The solution was to filter the light exiting the chamber with a 410 nm bandpass filter thereby obtaining relatively pure transmittance/absorbance.

#### Photoevaporation:

A second major complication was that continuous light stimulation by the LED produced sufficient thermal excitation that evaporation of the lysate/substrate mix was problematic. To minimize this, the light intensity was reduced by a neutral density filter and pulsed for 5 sec every min for a total of 15 min.

#### Magnetic system:

Initial prototypes using electromagnets to attract the paramagnetic beads had problems with voltage drain, slow attraction of beads, and heat production. Acceptable performance was obtained by attaching a static neodymium magnet (12 × 5 mm) to a rotating servo* motor that could rotate the magnet from a 25-mm distance to a 3-mm distance relative to the isolation chamber in <2 sec. Elastase rate constant (V_max_) calculation: The calculation of the elastase cleavage rate constant was performed by taking the raw blue (B) channel photometer readings and computing the % change from the 0 min reading to compute % Transmittance (% T). The % T was converted to absorbance by the formula: 2-LOG10 (100 * % T). The absorbance *versus* time curve was then fitted with a quadratic equation where the linear component (slope) is taken as the V_max_. This has the effect of minimizing any saturation of the curve at very high elastase activity. The program reports the goodness of fit as R2 with a typical empirical value of >0.99.

### Buffers and reagents

#### Bead Isolation Buffer (BIB):

BIB is composed of Hanks Balanced Salt Solution (HBSS) without calcium or magnesium Grand Island Biological Company (GIBCO), with EDTA and Bovine Serum Albumin (BSA) (5 μM and 1 mg/mL final concentrations).

#### Lysis Substrate Buffer (LSB):

Lysis buffer is Phosphate Buffered Saline (PBS) w/0.18 mM CaCl and 0.098 mM MgCl and Triton X-100 (0.2%, v/v, PH 8.0. The colorimetric elastase substrate, N-methoxysuccinyl-Ala-Ala-Pro-Val-p-nitro Anilide (N-AAPV-pNA, Sigma), was dissolved in 82% Dimethylsulfoxide (DMSO) to 6 mM and stored at −80 until use. The Lysis Substrate Buffer (LSB) is made by mixing N-AAPV-pNA elastase substrate with lysis buffer in a 7:3 ratios by volume (1.4 mM final concentration of substrate). For some select experiments, the fluorometric substrate, bis (N-benzyloxycarbonyl-L-tetra-alanyl) rhodamine, (CBZ-Ala-Ala-Ala-Ala)2Rh110, (Anaspec) was similarly prepared.

#### Antibody-coated paramagnetic beads:

Various antibodies to surface markers were examined, including CD15, CD16, CD11b, and CD66b. CD15 beads were purchased pre-conjugated from InVitrogen, while the others were conjugated with antibodies obtained as carrier-free purified antibody from Biolegend. Ultimately CD66b was chosen for because of the high and consistent yield. Antibodies were coupled to Dynabeads tosylactivated beads (Invitrogen) with minor modifications to the manufacturer’s protocol and resuspended in 225 ul aliquots of BIB. Purified human neutrophil elastase. Human neutrophil elastase (20–22 units/mg; Athens Research & Technology) was used to standardize our elastase measurements in the CyBIS device.

### CyBIS assay

Immediately following collection *via* fingerstick or venipuncture, 75 μl of whole blood is transferred and washed by an excess of BIB buffer (1 ml) and a low-speed centrifugation (300 g × 5 min). The plasma/BIB supernatant is then discarded, and the pelleted blood is resuspended in an aliquot of antibody-conjugated beads which is then transferred to the CyBIS chip. The chip is then inserted into the machine and the Luer inlet and outlet connectors are attached.

The core of the instrument is a housing that accepts the sample chip in a position so that an LED light source projects a 405 nm beam through the reaction chamber which is measured by a photometer (Panel A). The removable and disposable chip (Panel B) has Luer-type connectors to allow controlled fluid movement of buffers and substrates through a bottom reaction chamber that has borosilicate glass coverslip windows on each side to create the light path through the fluid. The supporting devices (Panel C), include an Arduino microcontroller that coordinates all operations, including the pumps, valves, LED, magnet, photometer, and touchscreen (Figure 1). The fluid operations (Panel D) are controlled by positive pressure from a small air pump (Bartels) that creates positive pressure on the reagent tubes containing buffers and substrate mixes, that are connected to the sample chip. The reagents are selected by a set of 6 small electrically-activated valves that direct the air pressure from the single air pump to the fluid reservoirs.

Once the assay is begun, the blood/bead solution is gently mixed to facilitate the binding of neutrophils to the paramagnetic beads using pulses of air (20 min). Following this mixing step, a neutral wash buffer (PBS no Ca++ or Mg++) is introduced to rinse unbound cells and non-cellular components of blood. A servo arm positions a neodymium magnet adjacent to the chip pulling the paramagnetic beads and bounded cells against the chip wall before the buffer is pumped in. The mixing process consists of resuspending the pelleted cells, repelleting with the magnet, and finally pushing out the supernatant and unbound cells and blood components. This process is then repeated to ensure the captured cells are thoroughly rinsed (3X total). A small quantity (400 μl) of LSB is then dispensed into the chip. The rinsed/retained bead-bound cells are lysed and the cleavage of the AAPV-pNA substrate by neutrophil elastase releases free Para-Nitro Aniline (PNA), which absorbs light strongly at 405 nm. With a 405 nm LED on one side, and a photometer on the other side, the absorbance created by the progressive cleavage to the elastase substrate is quantified over a 15 min period. Quadratic regression is then used to calculate the V_max_ of neutrophil elastase activity.

### Blood sampling of subjects

#### Consent:

This prospective, observational study, part of the sensor trial, was approved by the Institutional Review Board of the George Washington University (#NCR213645). All subjects provided their informed consent or, if the subject was incapacitated, their legally authorized representative provided consent on their behalf.

#### SIRS-positive Emergency Department (ED) patients:

Consenting patients presenting to the ED were selected based on having Systemic Inflammatory Response Syndrome (SIRS) scores of 2–4, indicating potential sepsis. SIRS criteria include a depressed or elevated White Blood Count (WBC) count, (<4000/ul or >12000/ul), fever or hypothermia (<36°C or >38°C), and cardiac or respiratory distress (heart rate >90 bpm, respiratory rate >22/min). Each of the characteristics above is scored one point for a maximum of 4 points, where 2 or more points is defined as SIRS-positive. Control patients. Control patients were recruited from consenting volunteers, including healthcare workers, staff, students and ED patients with no charted diagnosis of infection and a SIRS score <2, who self-reported being infection-free or with minor infections, such as the common cold.

#### Venipuncture:

Blood (4 ml) was obtained by standard venipuncture using a 21 g needle on a butterfly adapter connected to a BD Vacutainer K2 EDTA tube containing 7.2 mg of EDTA. All patient samples were assayed from peripheral venipuncture blood within about 30 min.

#### Finger stick:

For some laboratory optimization studies, small volumes of blood (75–150 μl) were obtained from a fingerstick with a BD Microtainer contact-activated lancet (2 mm) with the blood collected by a standard micropipettor and quickly transferred into the BIB buffer.

#### Blinding:

Laboratory technicians who processed the samples were blinded to the conditions of the patients by anonymous coding. However, the technicians’ processing included a White Blood Cell (WBC) count which constitutes a rough indicator of infection. Additionally, the WBC count is one of the four SIRS criteria gauging the risk of septic infection.

### Neutrophil isolation via surface marker affinity beads

Within 30 min post-collection, the neutrophils were isolated from the EDTA-treated blood sample using anti-neutrophil antibody-coated magnetic beads. Pre-washing of neutrophils was determined to be necessary to maximize the number of cells captured. EDTA-treated blood (75 μl) was diluted with an excess of BIB (1 ml), mixed, and then centrifuged (300 g × 5 min), and then 950 μl of supernatant (plasma and BIB), was discarded. The gently pelleted blood was then mixed with an aliquot of anti-CD66b-coated beads. Cell capture and washing were performed using neodymium magnets (12 mm diameter, 5 mm thick) for 2 min. Cell counts were performed using 10 μl in a hemocytometer viewed at 200X using phase contrast. The identities of the isolated cells were confirmed by fixing with 4% formaldehyde in PBS, followed by a May-Grünwald stain (Wright-Giemsa stain with eosin Y).

### Elastase assay

#### Manual method:

Neutrophils were isolated as described above. Aliquots of 75 μl blood containing 100,000–600,000 cells were used fresh. As the final step, the bead/cell aggregate was magnetically attracted to the tube wall, the supernatant removed, and the bead/cell mix was lysed with 140 μl of LSB containing PBS, Triton X-100 and elastase substrate. Substrate cleavage was quantified by reading the absorbance at 405 nm with a VersaMax plate reader at the specified time intervals.

#### Automated CyBIS method:

Essentially the same reaction was recreated in the small CyBIS reaction chambers starting with 75 μl of blood and anti-neutrophil paramagnetic beads. After incubation and washing, as described above, the captured cells were dissolved with LSB (PBS with Triton X-100 and the N-AAPV-pNA substrate). Absorbance at 405–410 nm was read at 1 min intervals for 15 min using a digital photometer connected to the Arduino.

#### Comparison of neutrophil surface markers for affinity enrichment:

CD15, Sialyl Lewis X, is a common surface marker for neutrophils, but it is reliant on a post-translational modification, and we observed that some patients were nonreactive. The CD66b (CEACAM8) surface marker captures both CD10^+^ mature neutrophils and CD10^−^ immature neutrophils [9]. Likewise, low density granulocytes are also CD66b^+^, and are known to produce high levels of NETs during short term *ex vivo* culture [10]. Magnetic isolation of neutrophils is found to cause less activation than density gradient methods [11,12]. However, activated neutrophils can release a soluble form of CD66b which could block antibody binding to cells and so the whole blood (75 μl) was diluted with BIB (1 ml), pelleted at 300X g, and 950 μl of supernatant was discarded prior to incubation of the cells with CD66b coated Dynabeads (prewashed) [13].

### Whole blood cell count and capture yield calculation

#### Cell counting:

The number of white cells in a sample was quantified by commercial reagents that rapidly lyse the Red Blood Cells (RBC) and stain the White Blood Cells (WBC) prior to physical counting in a hemocytometer. WBC counts were achieved by adding 20 μl of whole blood to a Leukotic tube (Bioanalytic), following the product’s protocol, ensuring the cells remained homogenously suspended, and counting the preserved cells within 2 h in a hemocytometer at 200X. Counting of cytocaptured cells was likewise achieved by counting in a hemocytometer. The percent yield of captured cells is expressed as captured/total*100.

### Optimized cytocapture and elastase assay program

Through a variety of optimization studies, the preferred parameters of the automated assay were identified and programmed into the CyBIS device.

#### Pre-wash:

As noted, using manual protocols, the need for prewashing of the neutrophils was identified and incorporated into both the CyBIS and manual protocols.

#### Blood/bead mix:

Largely using manual cytocapture protocols, the type of antibody, bead concentration, time and mixing method were optimized.

#### Capture and wash 3X:

Using both manual and automated protocols, the number of washes, volume and agitation level were determined to yield the highest number of intact neutrophils

#### Neutrophils:

**Figure 2:**
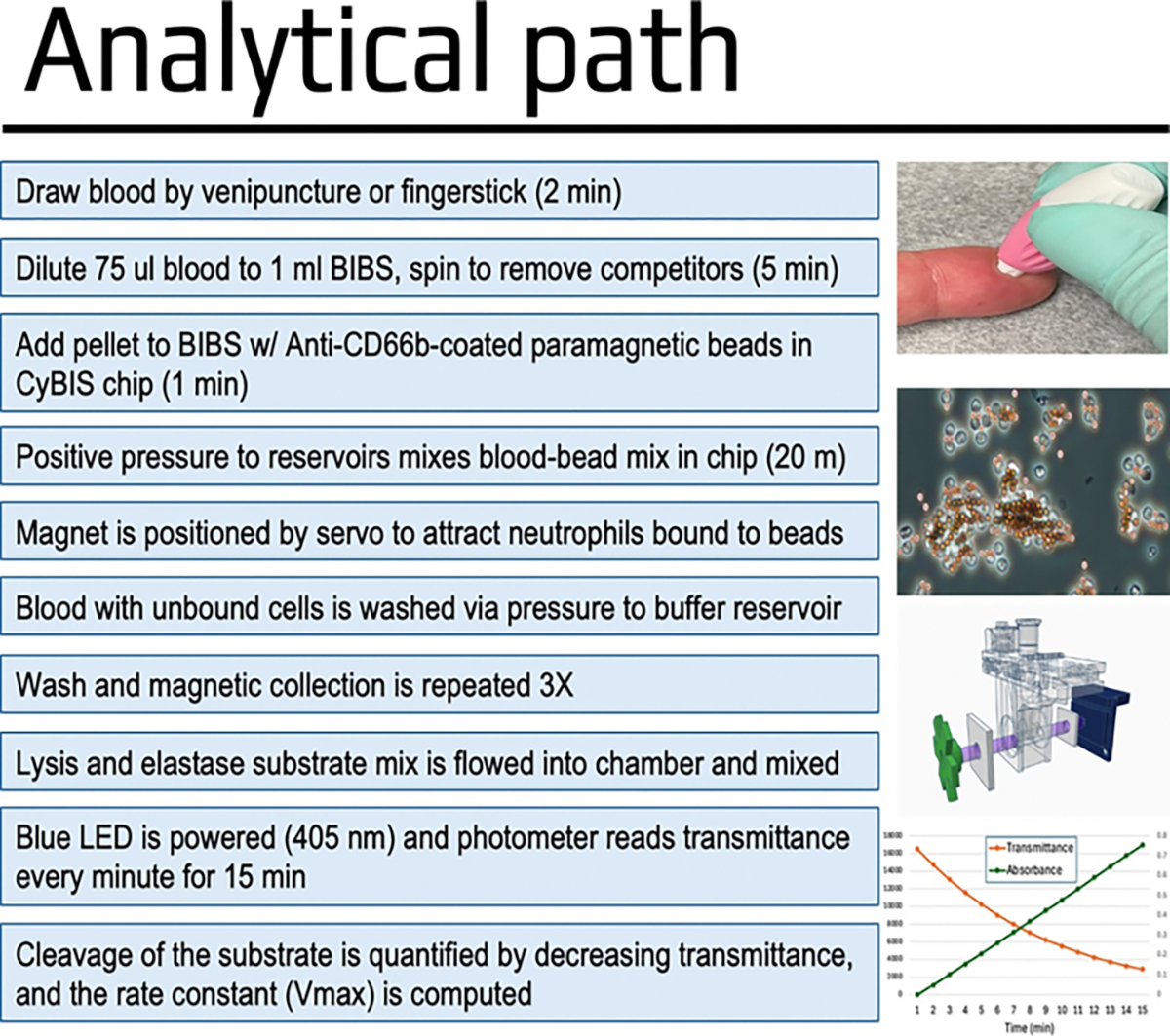
The analytical workflow for automated cytocapture and elastase assay. The basic steps from blood draw or finger stick, through obtaining the kinetic elastase results are outlined.

At very high agitation, there was poor binding and cell damage was evident, while at low agitation, there was poor yield of neutrophils. At a moderate mixing rate, created by alternating pressures on the inlet and outlet ports, intact neutrophils were isolated, typically with 2–4 beads per cell Figure 2.

#### Lysis and substrate mix:

While previously reported methods 5 achieved neutrophil lysis with repeated freeze-thaw cycles, that method was unsuitable for a POC device. It was found that a solution containing a detergent (Triton X-100) with the elastase substrate provided adequate cell lysis while preserving elastase activity.

#### Kinetic assay of substrate cleavage:

Both manual and automated studies identified the optimal light intensity, time and photometer settings to detect the cleavage kinetics of the elastase substrate.

## RESULTS AND DISCUSSION

### Comparison of surface markers for neutrophils useful for affinity enrichment

**Figure 3:**
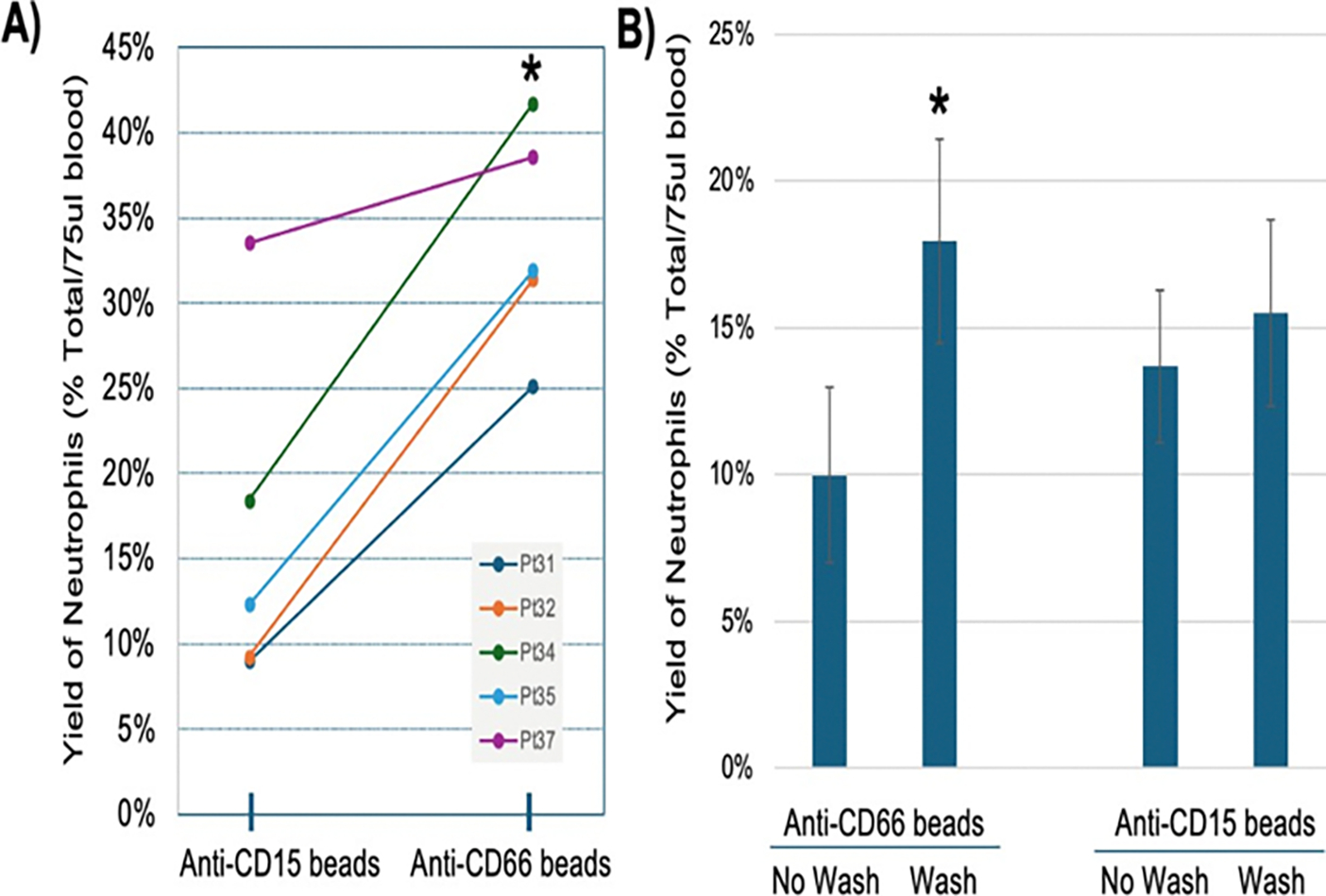
A) Human blood samples (75 μl) were prewashed and then incubated in BIB buffer with antibody-coated paramagnetic beads (antiCD66b or anti-CD15) for 20 min and then the beads and bound cells were magnetically retained in the tube while unbound cells and fluids were washed away 3 times. The number of retained cells was counted visually on a hemocytometer at 200X. In a set of 5 patients (Pt31, Pt32…), prewashed cells were isolated with anti-CD15 (left) or anti-CD66b (right). The % of cells recovered is shown on the Y axis; B) Blood samples were split and cells captured by 2 methods. In one method, the beads were added to whole blood (No Wash), or the blood cells were diluted with 1 ml of buffer and then centrifuged to remove soluble inhibitors of antibody binding to cells (Wash). Note that different patients were used for CD66b (n = 15) or CD15 (n = 7) isolations and thus the yields are not comparable between antibodies in this panel. * indicates p<0.05.

While CD15 has been commonly used as a surface marker selective for neutrophils, recent data has shown that it’s abundance on neutrophils from blood or saliva is relatively low compared to CD66 and other markers [14]. Using a commercially available anti-CD15 paramagnetic bead to isolate neutrophils from a series of ED patients and healthy control patients, some individuals yielded very low numbers of neutrophils, and this could not be attributed to a soluble inhibitor of CD15. Rather, some people seemed to lack the specific epitope for this particular CD15 IgM antibody clone MMA, and it appeared to be a reproducible feature of that person, as opposed to a technical defect. Thus, another isolation target was sought and initial studies using the manual isolation method indicated that CD66b (CEACAM8) was a better target. As shown in Figure 3, when CD66b antibodies were immobilized on the paramagnetic beads, the overall yield of cells was significantly higher than anti-CD15 bead yield by an average of 2-fold: Anti-CD15=16.5% (4.6% s.e.m.) *versus* anti-CD66b=33.7% (2.9% s.e.m.), p=0.003, n=5 pairs.

### Plasma contains inhibitors of CD66b antibody recognition of neutrophils

A major concern was that while a given marker might work well on normal subjects, it is possible that in inflammatory/infectious states, particular surface markers might be cleaved into a soluble competitor or masked by a circulating binding protein. CD66b (CEACAM8) is a granulocyte-specific activation marker upregulated on neutrophils during bacterial and viral infections [15]. CD66b engagement is thought to prime neutrophils and favour release of IL-8 from intracellular storage. CD66b has a soluble form that could interfere with anti-CD66b-coated beads [16,17]. Thus, we examined whether pre-washing the neutrophils increased their yield when isolated with the antibody-coated paramagnetic beads.

In a subset of patient blood samples, the whole EDTA-anticoagulated blood was either diluted directly into beads with BIB, or briefly pre-washed by an excess (~20X) of BIBS and centrifugation (300 g) to recover all cells prior to bead incubation. In all 7 patients analyzed for the prewash effect prior to CD66b bead isolation, a brief prewash increased the yield of cells from 63.8 cells/75 μl (10% yield unwashed) to 114.4 cells/75 μl (18% yield prewashed, p=0.014), presumably by removing inhibitors that block the antigen-antibody interaction. This effect was not observed in a different set of 16 patients isolated by CD15 antibody: CD15 unwashed=13.7% *versus* CD15 prewashed=14.5%, p=0.478 Figure 3B.

### Dose response of purified elastase

**Figure 4:**
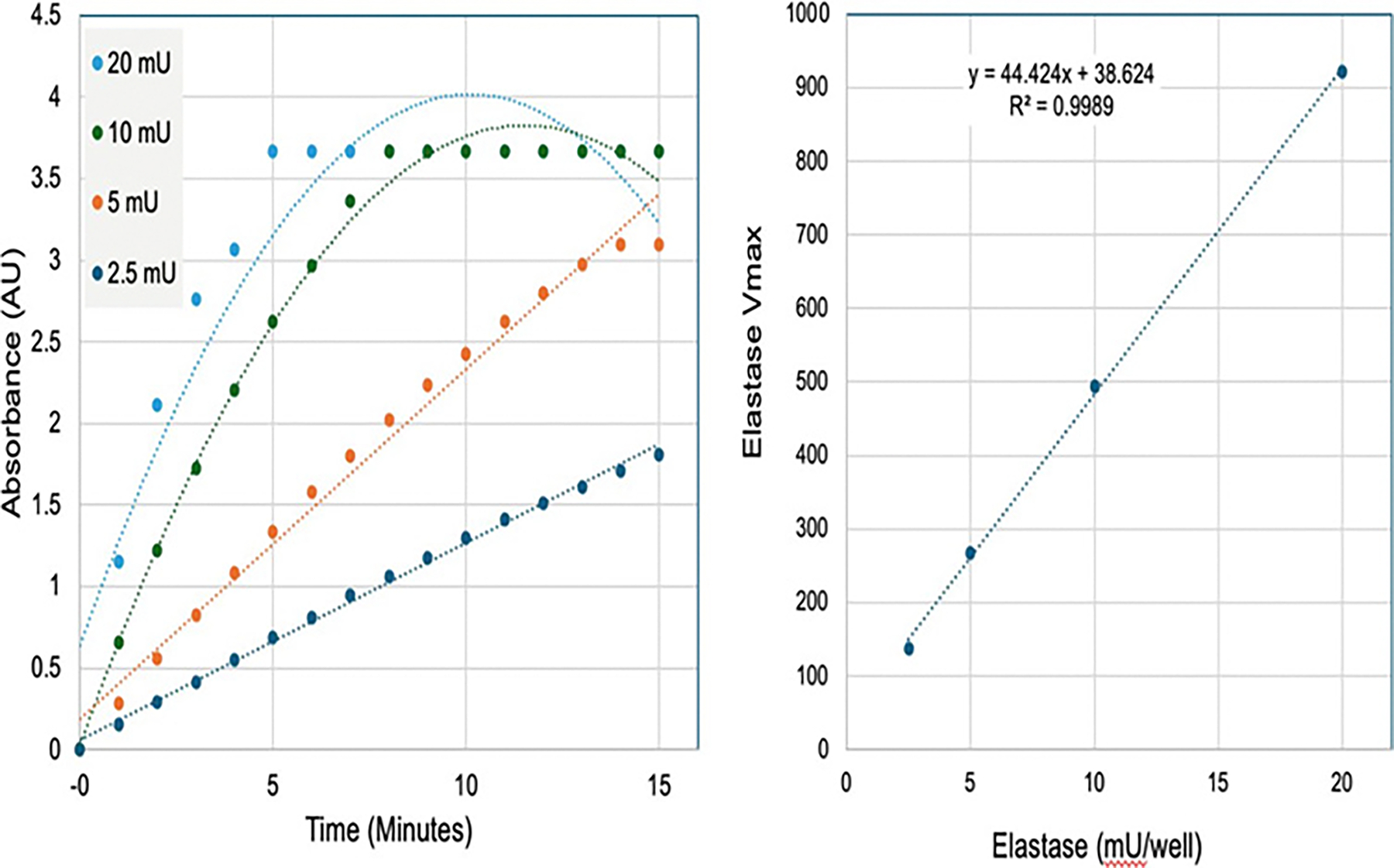
The effect of dose of purified elastase on the substrate cleavage rate in the CyBIS device. Note: A) Specified doses of purified human neutrophil elastase (0–20 mU) were reacted with the lysis buffer containing the elastase substrate and the absorbance was measured at 405 nm at 1 min intervals for 15 min. B) The computed rate constants (Vmax, y-axis) from the plots in Panel A are plotted against the concentration of elastase (mU, x-axis)

Having determined the optimal antibody for neutrophil isolation, and the necessity for prewashing of the neutrophils, we sought to determine how the CyBIS device would respond to a defined range of purified elastase. The reaction chamber was loaded with specified amounts of purified human neutrophil elastase added to the lysis/substrate buffer, and then read over the same kinetic interval (15 min). At lower levels of purified elastase (2.5–5 mU), the change in absorbance over time was essentially linear, while at the higher concentrations (10–20 mU), the absorbance saturated, as expected Figure 4A. This was readily corrected by using the linear component of the quadratic fit that yielded rate constants (V_max_) that showed stepwise increases with elastase dose (R2=0.9989), as shown in Figure 4B.

### Neutrophil numbers vs. elastase levels

Another strategy to test the instrument’s response was to vary the number of input neutrophils and measure the reported V_max_. This approach is more comparable to the clinical use of the instrument than measuring purified elastase because it would account for differences in the presence of elastase inhibitors that might be carried by neutrophils.

**Figure 5:**
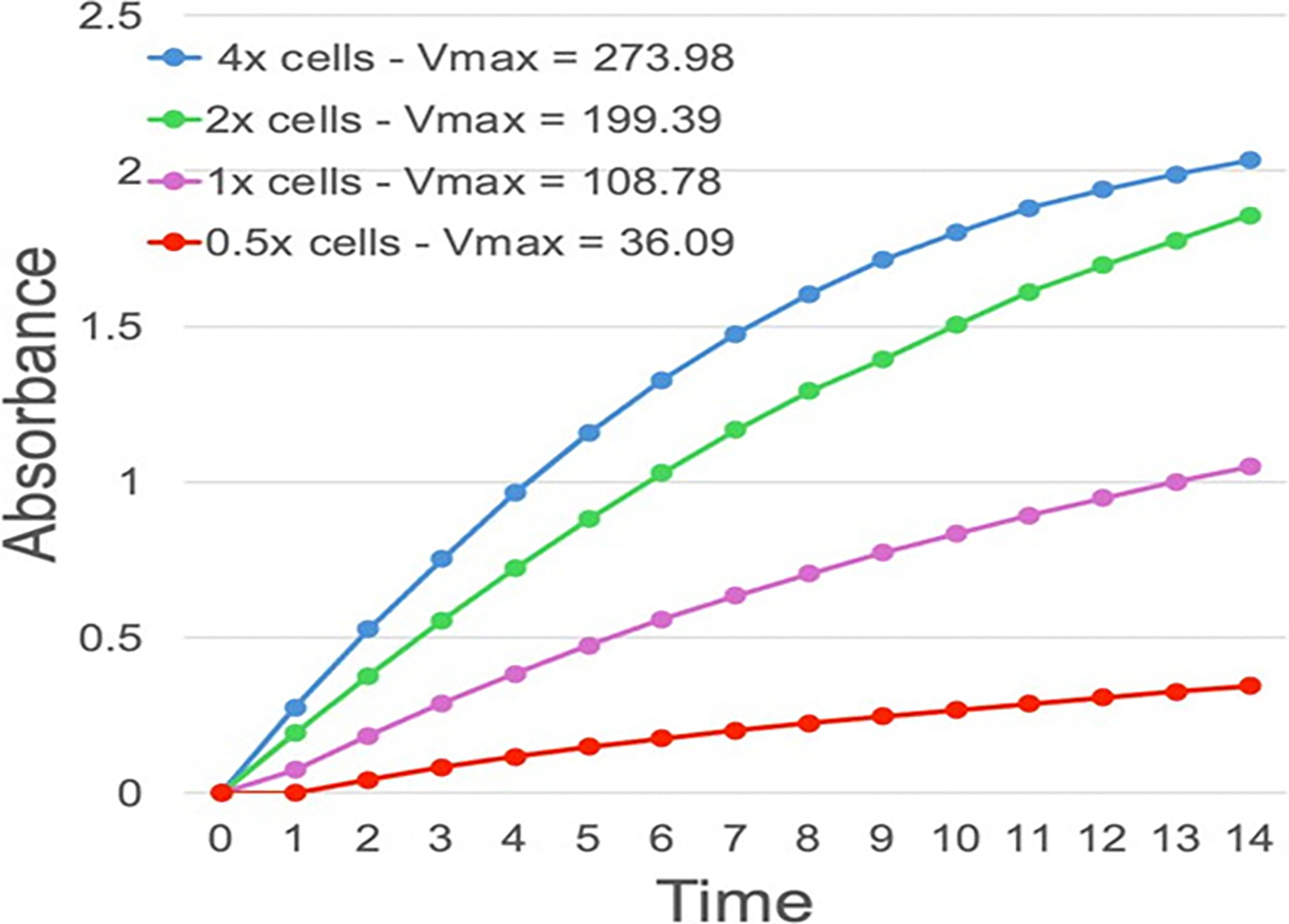
The effect of variable neutrophil numbers on elastase measurement in CyBIS. The effect of varying the number of cells analyzed in the CyBIS device was examined by isolating a large number of cells from one subject and then diluting the cells into a concentration range corresponding to known variation within typical human subjects (0.5–4X). After dilution, the cells were mixed with the lysis/substrate mix and quantified by CyBIS for elastase cleavage over time. The computed rate constants are shown in the figure legend.

Thus, a large number of neutrophils were isolated by CD66b beads and then they were diluted. As shown in Figure 5, the V_max_ reported by CyBIS showed a stepwise increase with increasing cell numbers over an 8-fold range (0.5–4X). There was some suggestion that the V_max_ saturated at the highest cell numbers used, and it is possible that the relationship between V_max_ and true elastase content might also benefit from a quadratic fit to minimize the effect of saturation at high levels. However, in actual use, a V_max_ of ~250 has only been seen in a single patient, and most normal subjects show a V_max_ in the 25–50 range.

### Neutrophil elastase activity is only partially dependent on cell number in patient samples

The relationship between the number of cells captured from different patients and the resulting V_max_ of elastase activity was examined (CD15 isolation). It was observed that the expected number of cells captured in the CyBIS was not well correlated to the V_max_ (r=0.22, n=30). Similarly, in a series of 27 samples with cytocapture by CD66b beads, the white cell count was only modestly correlated to the V_max_ (0.34). A caveat is that the number of cells captured within the CyBIS device could differ from the number captured by running the same sample in parallel by an identical manual method that is necessary for the cells to be physically counted on a hemocytometer. However, it remains likely that the V_max_ is the result of both the number of neutrophils and their elastase content, as observed more directly in our lab’s previous study where COVID-19 patients showed 5-fold higher elastase activity per cell [5].

### Instrument to instrument reproducibility

**Figure 6:**
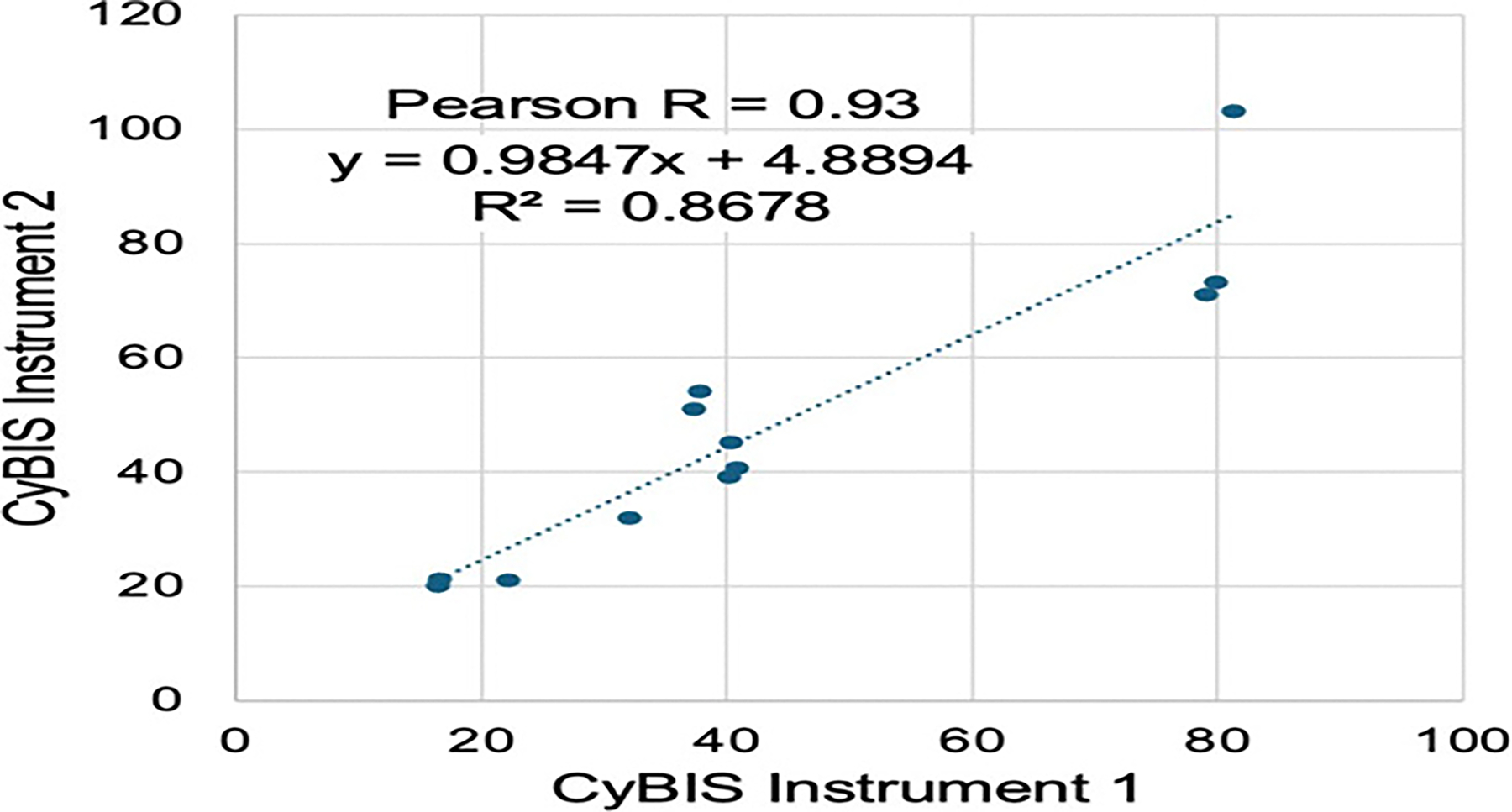
Reproducibility between instruments, two CyBis devices were built and calibrated to similar performance of flow rates and light intensities. Blood samples from 12 subjects, with or without known infections, were divided into 2 samples and then analyzed in parallel on the 2 instruments. Each point reflects the measured Vmax from the same sample on the 2 instruments. A linear fit (dashed line), Pearson R correlation and the R^2^ are reported.

Control and patient samples (n = 12) were divided into 2 identical samples immediately after the wash stage and then assayed at the same time in 2 separate CyBIS devices. As shown in Figure 6 the reproducibility between the devices is excellent, with a Pearson correlation 0.93. One instrument tended to read slightly higher than the other (Average V_max_=47.8 *versus* 43.5) but it was not statistically different by paired t-test (p=0.14).

### Elastase activity in SIRS-positive ED patients

**Figure 7:**
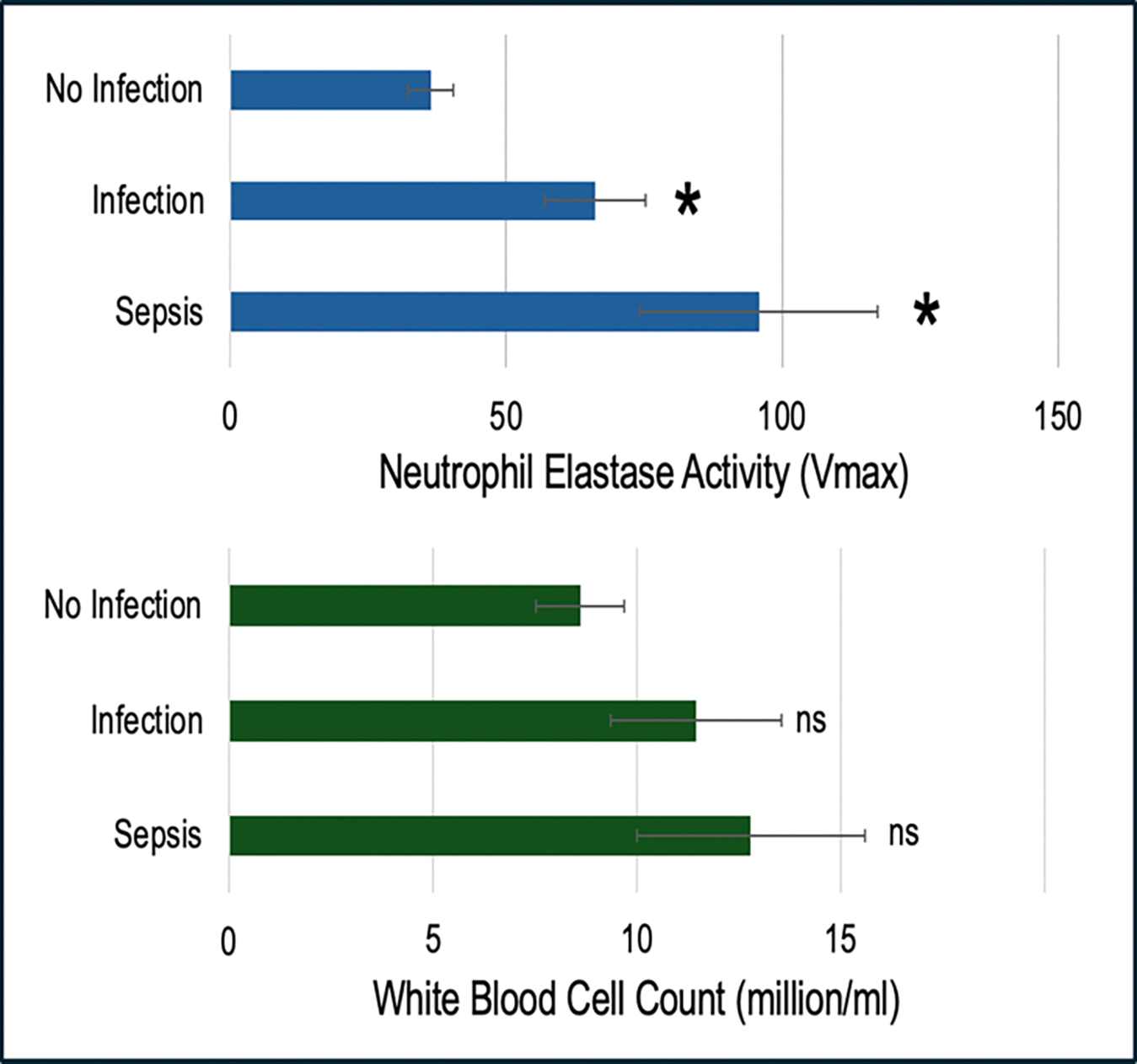
Neutrophil elastase activity by CyBIS and white blood cell count on subjects with varying degrees of infection. Blood samples from an ongoing clinical trial involving both CyBIS and a panel of RNA biomarkers of infection (The SENSOR Trial) were divided into groups based on a clinically adjudicated likelihood of No Infection (n=14), Infection (n=13), or Sepsis (n=9), defined as a bacterial infection reaching the blood stream and triggering a severe immune response. In the upper panel, average CyBIS Vmax levels are reported by group, and in the lower panel the white blood cell count is reported. *=p<0.05 relative to the No Infection group, ns=not significant relative to the no infection group.

The potential clinical utility of CyBIS was evaluated on samples collected during an ongoing clinical study of patients presenting to the ED and exhibiting clinical signs of potential sepsis. Many of the subjects were ultimately found not to have infections or sepsis, while in others an infection or sepsis was determined with high confidence. In about 5%−10% of patients there was evidence of sepsis, whereby the infection reached the bloodstream at a sufficient level to trigger a rapid and life-threatening immune response. Based on a 30-day post-sampling chart review the patients were categorized into the 3 groups shown in Figure 7. The CyBIS V_max_ showed a stepwise increase in average elastase neutrophil activity of 36.6 in no infection cases to 66.1 in cases with infection (p<0.005), to 95.8 in patients with clinical signs of sepsis (p<0.005 *versus* No Infection, p=0.086 *versus* Infection). The same patients were confirmed for infection status using RNA biomarkers of neutrophil activation quantitated by droplet digital PCR (not shown) [4–6].

The WBC count showed a similar trend of increasing counts with infection severity although it did not reach statistical significance. This finding is consistent with a wealth of data indicating that WBC count alone is a relatively insensitive measure of infection severity. For instance, meta-analysis indicates that WBC count has very modest sensitivity and specificity (both 73%), with only a 50% Negative Predictive Value (NPV) for diagnosing complicated appendicitis, which is the most common Gastro Intestinal (GI) infection seen in the Emergency Department (ED). This emphasizes the need for more advanced diagnostics, such as CyBIS, that measures the number and activity of neutrophils [18].

## CONCLUSION

An automated device such as CyBIS can provide rapid and accurate assessment of elastase activity present by isolating and assaying neutrophils from human blood. The CyBIS V_max_ is different than a WBC count because CyBIS measures the additive values of neutrophil abundance and their content of neutrophil elastase. The levels of neutrophil elastase activity may have diagnostic applications for human infections and sepsis, though larger clinical trials with the device are needed.

## LIMITATIONS

In the present format, CyBIS does not distinguish between an elevated neutrophil count *versus* a normal count with elevated elastase activity per cell. An improved version would be able to count the captured cells and then quantify elastase per cell by imaging.

This version of CyBIS uses relatively large volumes of blood (75 μl) and wash buffer (4.5 ml) per run, which could be reduced to 5 μl of blood and 300 μl of buffer in an integrated microfluidic design.

The current time from the sample input to the result is about 45 min, but by improving the mixing, washing, and reading steps, this could likely be reduced to ~15 min. For instance, while the elastase reaction is currently read for 15 min, a stable answer is available in 5 min.

## FUTURE DIRECTIONS

### Several improvements are foreseeable

- First, CyBIS could capture and image cells in a 2D format that would allow for cytocapture, counting of neutrophils and elastase activity quantified on a per cell basis.
- Quantifying elastase on a per cell basis is especially attractive because it is not known whether the elevated elastase activity results from an increase in all neutrophils, or more likely, reflects the influx of a subset of cells with elevated elastase activity.
- The fluid reservoirs and reagents could be built into the chip, which would reduce complications from imperfect connections and reduce distances for fluid movement.
- An internal control system to calibrate the assay, especially on the capture efficiency dimension, would be valuable.
- Presently, CyBIS operates at ambient temperature, but in field-deployed settings, either temperature control and/or temperature compensation would be important features.
